# Smart Monitoring of Manufacturing Systems for Automated Decision-Making: A Multi-Method Framework

**DOI:** 10.3390/s21206860

**Published:** 2021-10-15

**Authors:** Chen-Yang Cheng, Pourya Pourhejazy, Chia-Yu Hung, Chumpol Yuangyai

**Affiliations:** 1 Department of Industrial Engineering and Management, National Taipei University of Technology, Taipei 10608, Taiwan; cycheng@ntut.edu.tw (C.-Y.C.); claire830110@gmail.com (C.-Y.H.); 2 Department of Industrial Engineering, UiT The Arctic University of Norway, Lodve Langsgate 2, 8514 Narvik, Norway; pourya.pourhejazy@uit.no; 3 Department of Industrial Engineering, School of Engineering, King Mongkut’s Institute of Technology Ladkrabang, Bangkok 10520, Thailand

**Keywords:** smart monitoring, signal analysis, overall equipment efficiency (OEE), intelligent decision-making

## Abstract

Smart monitoring plays a principal role in the intelligent automation of manufacturing systems. Advanced data collection technologies, like sensors, have been widely used to facilitate real-time data collection. Computationally efficient analysis of the operating systems, however, remains relatively underdeveloped and requires more attention. Inspired by the capabilities of signal analysis and information visualization, this study proposes a multi-method framework for the smart monitoring of manufacturing systems and intelligent decision-making. The proposed framework uses the machine signals collected by noninvasive sensors for processing. For this purpose, the signals are filtered and classified to facilitate the realization of the operational status and performance measures to advise the appropriate course of managerial actions considering the detected anomalies. Numerical experiments based on real data are used to show the practicability of the developed monitoring framework. Results are supportive of the accuracy of the method. Applications of the developed approach are worthwhile research topics to research in other manufacturing environments.

## 1. Introduction

Business benefits from the transformation to smart manufacturing due to improving product quality and responsiveness to customer demand at a lower operational cost [1]. This transformation requires a certain level of operational flexibility, adaptability, and intelligence [2]. As one of the major enablers of smart manufacturing [3], Cyber–Physical Systems help facilitate process intelligence by connecting the virtual, information world with the physical environment in which the operations take place [4]. Through this connection, Cyber–Physical Systems provide decision support with detecting deficiencies, adjusting the operational parameters, and, more generally, providing operational flexibility [5,6]. Effective communication between machines and intelligent decision-making is the main tenet to take full advantage of Cyber–Physical Systems in smart manufacturing systems [7].

Automated operations require process transparency enabled by effective monitoring of the tasks and integration of the involved Man, Machine, Material, and Methods [8]. Intelligent decision-making facilitates this integration across the operating network through the flow of information and hardware and software [9]. In this situation, collecting real-time data from the operating network, providing analytics-based feedback, and computational capability for the adjustment of operational properties, so-called smart monitoring is necessary [10,11]. Intelligent decision-making requires real-time access to operational data and computationally efficient analysis frameworks, which are not applicable using traditional data collection approaches.

Monitoring the state of a machine and the production process for fault detection have been traditionally carried out manually, resulting in significant operational inefficiencies and flaws in the flow of the information [12]. Intelligent decision-making in modern manufacturing highlights the need for advanced data collection technologies, where the real-time acquisition of data on production status is principal [13]. Programmable Logic Controller (PLC) is used to monitor internal operations, such as sequence control, timing, and counting, without human interactions. PLCs are reliable, versatile, and simple to implement and have seen significant development in the academic literature. Reference [14] developed a PLC system for monitoring the characteristics of eggshells to effectively identify nonstandard eggs. Reference [15] suggested integrating PLCs into engines to prevent hardware and software failures through monitoring production speed and efficiency. Reference [16] applied neural networks to monitor PLC programs and predict PLC errors, which improves the fault detection capabilities, maintenance, and reduces downtime of the production line. Reference [17] applied a PLC-based approach to monitoring renewable energy generation by collecting information on energy consumption. Despite its merits, PLC-based approaches cannot provide the details on the status of a given machine or specific items without the help of sensors.

Once an anomaly is detected by sensors, the underlying causes should be investigated [18]. The mere use of historical data-based approaches may negatively impact the time-critical aspects of monitoring [19], and result in resource wastage and other difficulties, like increased processing failure, poor product quality, and operational disruptions [20,21,22,23]. Sensors and signal processing tools have been employed to, respectively, read/collect machine data and analyze it for real-time monitoring of manufacturing systems [24,25,26]. Real-time fault detection systems have been successfully employed in the literature [27,28,29,30]. Despite the importance of computational efficiency and robustness of signal processing in real-time fault detection, it received relatively limited attention in the smart monitoring literature. In the most relevant studies, reference [31] developed a statistical approach based on signal classification using Bayesian Naïve Classifier and simulations for condition-based maintenance [31], which assumes that states are independent. Reference [32] developed a condition monitoring framework based on compressed signal processing for diagnosing the machinery faults in real-time. They used a feature extraction method for analyzing signals collected by vibration sensors. These methods cannot distinguish signals received within the production process and provide Overall Equipment Efficiency (OEE)-based feedback. Our study extends to address this gap by developing a novel multi-method framework. For this purpose, the Wavelet Transform (WT), Hilbert–Huang Transform (HHT), Fast Fourier Transform (FFT), Hidden Markov Model (HMM), and Dynamic Time Warping (DTW) are integrated for analyzing machinery status signals retrieved from noninvasive sensors. The proposed approach facilitates a “plug-and-play” monitoring system enabling intelligent decision-making.

The rest of this manuscript begins with a background on data collection and analysis methods in the manufacturing context. Section 3 elaborates on the proposed multi-method framework. Section 4 presents the experimental settings and a discussion of the results and the implications. Finally, the concluding remarks and suggestions for future research are provided in Section 5.

## 2. Background

OEE is one of the most commonly used performance indicators for monitoring manufacturing systems; it is particularly useful for measuring the actual and theoretical production capacity and the analysis of operational performance. OEE helps identify the cause of equipment-related anomalies, where early failure detection brings significant cost reduction. OEE also has implications for minimizing waste and manufacturing costs, i.e., tracing the quantity of poor-quality products [33] and improving productivity. Besides, monitoring the extent of wear and tear in different parts of equipment using OEE maximizes resource utilization within the Total Productive Maintenance context [34]. OEE can also be treated as a random variable to overcome the drawbacks of traditional machine monitoring, enabling the analysis of dynamic environments [35]. Overall, monitoring the operational state of all equipment and eliminating OEE-independent conditions help provide more realistic measures [36]. Advanced data collection and analysis methods are the critical elements of smart monitoring for intelligent decision-making.

### 2.1. Data Collection Using Sensors

Sensors can be classified into invasive and noninvasive [37]. Proximity sensors, radioactive sensors, and vision sensors are prime examples of invasive sensors that enable instant and accurate monitoring. Proximity sensors monitor the distance between the tool edge and the product [38,39]. Radioactive sensors have been used to measure tool wear [40,41]. Vision sensors have been employed to measure the distance between manufacturing tools and their targets [42]. Noninvasive sensing devices, like vibration sensors, force sensors, and acoustic emission sensors are mainly designed to read the parameters associated with product and machining tools. Advanced force sensor as a dedicated reading device to identify target objects [43]. Miniaturized force sensors are used as microelectromechanical systems for application in mobile panels [44,45]. Vibration sensors have been applied to detect human breathing frequency to monitor the physiological condition of the human body [46]. Vibration sensors are also used to monitor the fatigue of offshore wind turbines [47]. From other notable application areas, sound waves are employed to sense voltage changes, which significantly reduces the risks in voltage measurement [48]. Finally, acoustic emission sensors have been employed for fault detection and the classification of large machinery [49]. The next section reviews the seminal methods developed for processing and analysis of the automatically collected data from the manufacturing equipment.

### 2.2. Data Analysis Methods

Information visualization for factory performance analysis helps the managers to effectively monitor the operations through reformulating complex issues into simple indicators [50], like equipment availability and product quality. Various techniques have been developed for analyzing collected manufacturing data, i.e., signals. The WT-based methods are suitable for analyzing high-frequency special signals or signals that change slowly over a long period [51]. The Fourier Transform (FT) method is effective for analyzing linear systems and the situations with the frequency components of a continuous waveform [52]. The HHT is a time-domain signal analysis method that is widely used to analyze nominal characteristics [53]. The DTW methods facilitate matching signals considering their similarities [54]. These works are reviewed below.

WT method consists of applying a series of transformations to instantaneously examine the time domain of the original signal as well as the frequency domain [55]. In this approach, the fundamental wave amplifies at low frequencies and yields time-domain information. In doing so, the characteristics of elastic time and frequency can be effectively displayed. Besides, the fundamental wave gets thinner at high frequencies and yields frequency-domain information. More recent studies proposed to integrate a Binary Pass Filter Processing into the WT method to decompose signals oscillating in time and frequency [56]. For this purpose and considering discrete WT, [57,58] proposed Equations (1) and (2) to group the sampling signals, $S=\left(S_{1},S_{2},\ldots ,S_{n}\right)$, into a number of approximate signals, $a_{n}$, and n detailed signals, $d_{j}$. In this approach, the reversible calculation of the WT and inverse WT can be expressed mathematically in Equations (3) and (4).

(1) $$f\left(d_{j}\right)\in \left[2^{-\left(j+1\right)}f_{s},2^{-j}f_{s}\right]$$

(2) $$f\left(a_{n}\right)\in \left[0,2^{-\left(n+1\right)}f_{s}\right]$$

(3) $$W\left(a,b\right)=\frac{1}{\sqrt{a}}\int_{-\infty }^{+\infty }f\left(t\right)\psi \left(\frac{t-b}{a}\right)dt$$

(4) $$f\left(t\right)=\frac{1}{c}\int_{-\infty }^{+\infty }\int_{-\infty }^{+\infty }\frac{1}{a^{2}}W\left(a,b\right)\frac{1}{\sqrt{a}}\psi \left(\frac{t-b}{a}\right)\begin{array}{cc} da & db \end{array}$$

where W represents the wavelet coefficient, a,b, and c refer to the scale parameter, translation parameter, and a constant value, respectively. Extending this seminal work, [59] developed a discrete WT for the monitoring and early fault detection of asynchronous motors, and [60] used a WT-based diagnostic method for fault detection in three-phase wound rotor motors.

The FT method decomposes the signal into several Sine wave frequencies, i.e., the summation of several Sine functions. Given the practicality of this feature, the FT method is often applied in spectral analysis [61]. In their proposed approach, Equation (5) is used to calculate $F\left(\omega \right)$ as the distribution of the time function $f\left(t\right)$ in the frequency domain and $e^{\left(-j\omega t\right)}=\cos\left(\omega t\right)-j\sin\left(\omega t\right)$.

(5) $$F\left(\omega \right)=\int_{-\infty }^{+\infty }f\left(t\right)e^{\left(-j\omega t\right)}dt$$

Considering infinity in the integral range of the time function, the signal should be converted to the frequency domain, which may result in partial loss of the time-domain information. Reference [62] suggested integrating FFT and WT for vibration signals, demonstrating that the time-frequency features can be used to obtain fault characteristics. As an alternative approach, [63] combined principal component analysis with the FFT method to classify and analyze brainwave signals. Given that fault signals contain noise and other interferences; complications may arise when rendering the characteristics data. To address this issue, effective noise filtering is a much-needed tool for machinery fault diagnostics. Besides, signals indicating a mechanical failure are usually nonlinear and nonstationary. The Empirical Mode Decomposition (EMD) developed by [64] is an adaptive signal analysis method that decomposes complex vibration signals into several inherent modal functions (IMFs) to address the above-mentioned issues. In this approach, each IMF characterizes a single shock signal component with only one frequency at a given time after decomposing. In doing so, the time-frequency distribution of the complex signals can be defined. To construct the time-frequency energy distribution, a different frequency calculation method can be used to obtain IMFs’ instantaneous frequency and amplitude, resulting in Hilbert Time Spectrum (HTS). Incorporated with time, HTS acquires the frequency–energy relationship plot, i.e., the Marginal Hilbert Spectrum (MHS). In MHS, different IMFs correspond to various signal characteristics, where faults can be detected through signal characteristic analysis. Reference [65] extended the EMD method for decomposing faulty power signals that categorizes the power failure signals of different sections considering the anomalous segments on the Hilbert time-frequency diagram. The HHT method was also applied to convert the vibration signal decomposition into diagnose faults [66], showing that its extraction feature can be used to inspect the characteristics of various fault situations.

The DTW methods are usually used for exploring time series data and dynamic programming to determine the similarity considering the different lengths. For this purpose, the similarity between time series is calculated using Euclidean Distance between the starting point $D\left(1,1\right)$ and the endpoint $D\left(m,n\right)$; the resulting value is used to find the shortest path as a gauge to determine similarity. This method has been widely used in various application areas, like speech recognition where the speed of speech varies but the signal is constant. Reference [67] applied the Mel Frequency Cepstral Coefficient (MFCC) for analyzing voice messages where the DTW method is used to compared voice signals from an external environment with those in the database. Reference [68] introduced the unbounded-DTW method that identifies the possible matches between two sequences using a fixed range for the forward and backward calculations and a minimum length, $L_{min}$. In this approach, unimportant similarities are ignored and the computational procedure can begin from any comparison point; it results in shorter average steps to meet the conditions for determining the path, hence, shortens the computational time. Besides, the U-DTW method is different from DTW in that it applies a Cosine Function, $\cos\left(\theta \right)=\frac{m\cdot n}{\mathrm{||m||}\mathrm{||n||}}$, to determine the similarity between two vectors, m and n, considering the angle θ between them. Applying this approach in the speech matching application area, [69] showed that U-DTW outperforms DTW.

**Figure 1:**
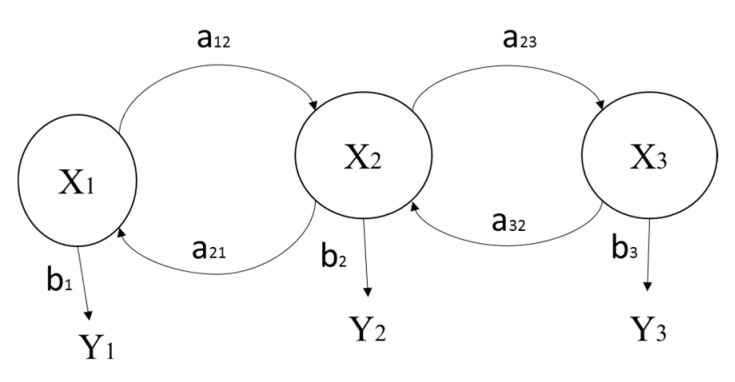
Conceptual diagram of the Hidden Markov Model.

Finally, the HMM applies to known information to detect unknown states, which is effective for determining the state of hidden signals [70]. To identify hidden or unknown states, $X_{1},X_{2},X_{3}$, the observed parameters that are impacted by these values are considered following the concept shown in Figure 1. In this approach, the output associated with $b_{1},b_{2},b_{3}$ probabilities can be used to observe the state of the information if there exists a probability distribution $a_{12},a_{21},a_{23}$. Reference [71] employed the MFCC method to decompose the speech signals of different words to enter heterogeneous HMMs; they showed that a higher accuracy can be achieved with more HMMs. The developed framework in the next section combines the advantages of WT, HHT, FFT, and HMM to distinguishing production signals and provide OEE-based monitoring for intelligent decision-making.

## 3. Proposed Framework

**Figure 2:**
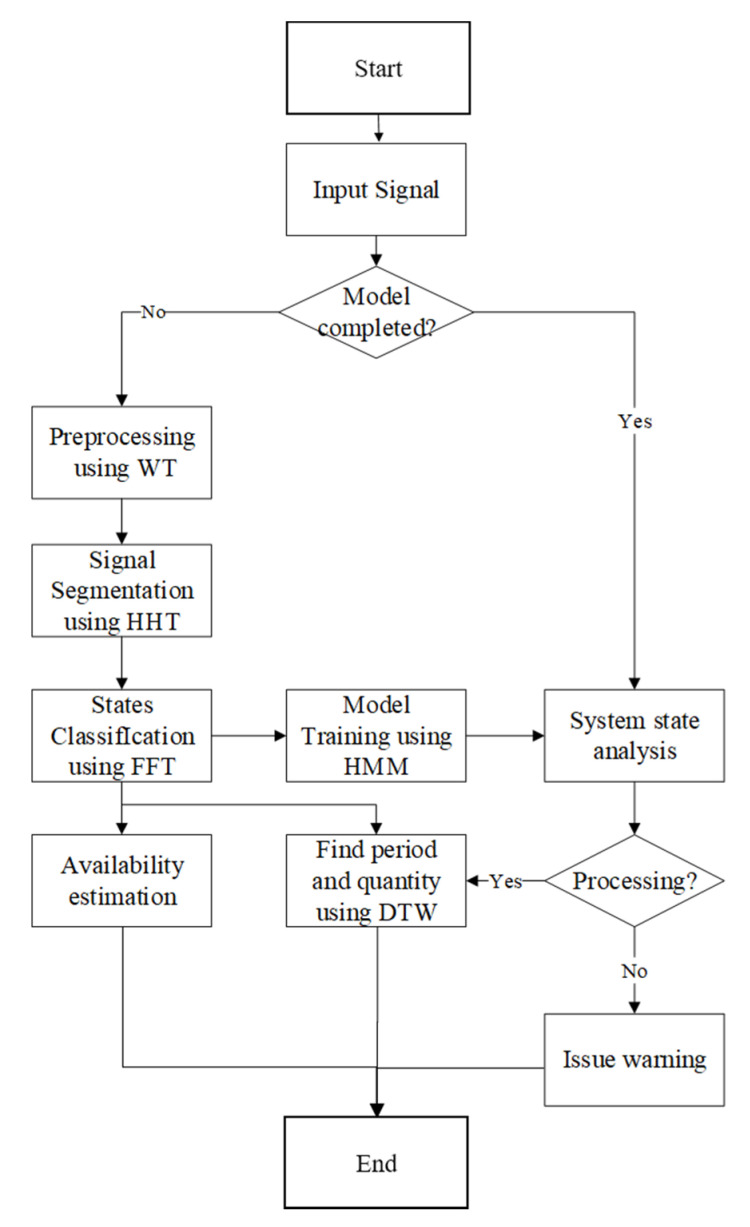
Flowchart of the proposed framework.

This research proposes to integrate signal analysis and information visualization to facilitate the monitoring of manufacturing equipment for increasing accuracy and computational efficiency. The three-step signal processing framework is shown in Figure 2. In this method, the input signal is pre-processed before constructing a model. The signal is then segmented and analyzed to identify the machinery status based on the characteristics of the signals. Calculating the ratio of processing and non-processing times, machine availability can then be examined. Next, the states are trained separately comparing them with the signal status. Finally, the processing status is determined, the production quantity is calculated, and the manager is notified to take well-informed actions in the corresponding stage.

### 3.1. Step 1. Pre-Processing of the Input Data

**Figure 3:**
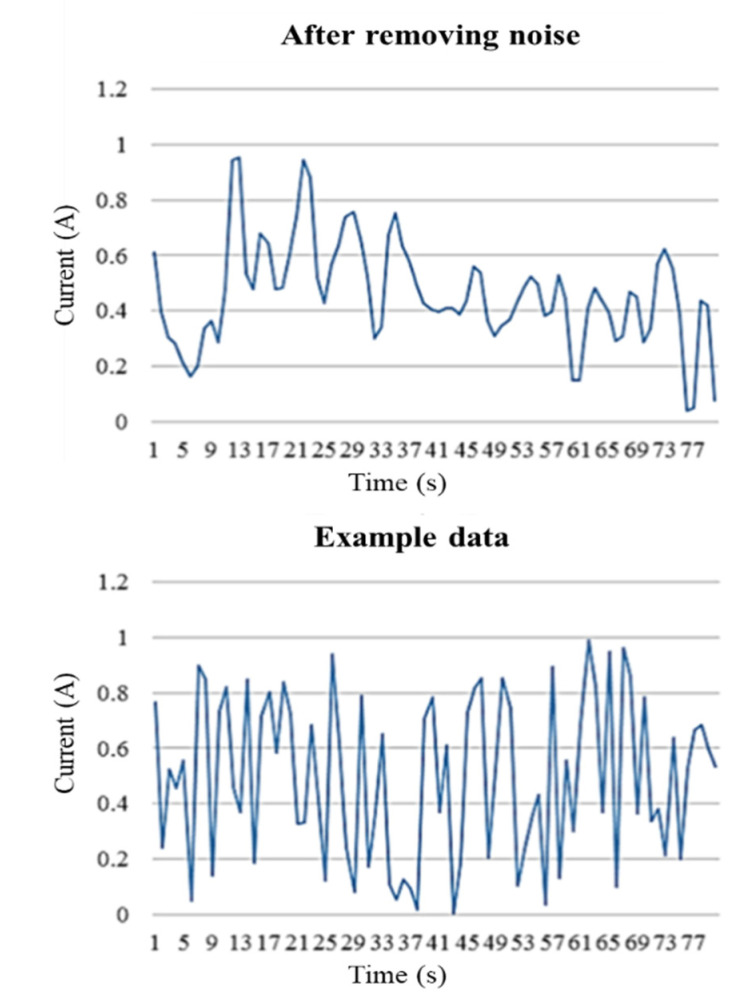
Filtering of a Wavelet Transform.

WT is used to minimize noise and maximize the retention between the main features and the original signal. Applying this approach, the main wavelet function is translated and scaled, which shows either a high-frequency in a short period or a low-frequency with a long period. This method is particularly suitable for determining high-frequency special signals or those that change slowly over a long period. On this basis, frequency- and time-domain analyses are applied to filter the signal and noise coefficients characterized by different features and construction rules in different scales. By so doing, the signal can be effectively distinguished from noise. Once the wavelet coefficients are obtained, they are processed nonlinearly and the inverse WT is applied to scale back the signal. The filtering results are shown in Figure 3.

### 3.2. Step 2. Signal Segmentation Considering Technical Characteristics

**Figure 4:**
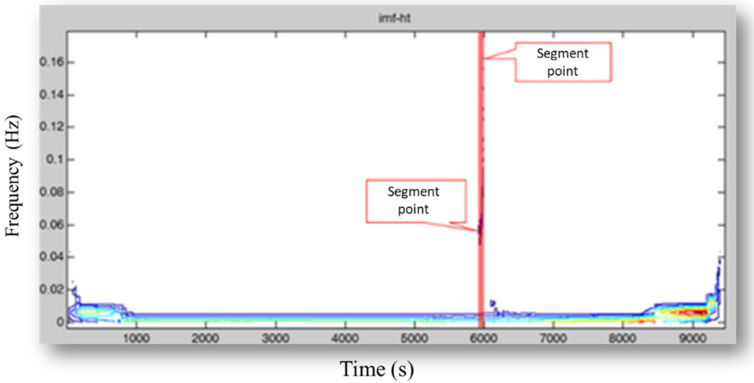
Hilbert’s time-frequency chart.

The HHT, as an adaptive time-domain signal analysis method, is applied to segment the filtered signals. The signal decomposition of different bases results in varying outputs. The HHT improves the non-steady and non-linear status signal analysis performance. Figure 4 demonstrates an illustrative example of Hilber time-frequency that helps determine the characteristics of each signal segment. Although the HHT chart enables the analyses of mixed-status signals, it may result in flattening the non-processing signals and dismissing the signals with fewer fluctuations. To address this shortcoming, this study suggests including two constraints, Equations (6) and (7), to determine whether to continue the segmentation. It helps prevent dismissing the non-processing signals. In this approach, $max_{new}$ and $max_{old}$ represent the absolute value of the maximum slope after and before segmentation, respectively. *α* and *β* are the respective ratios.

(6) $$\alpha \times \max_{new}<\max_{old}<\beta \times max_{new}$$

(7) $$\max_{old}>\beta \times max_{new}$$

### 3.3. Step 3. Output Analysis and Training of the Model

**Figure 5:**
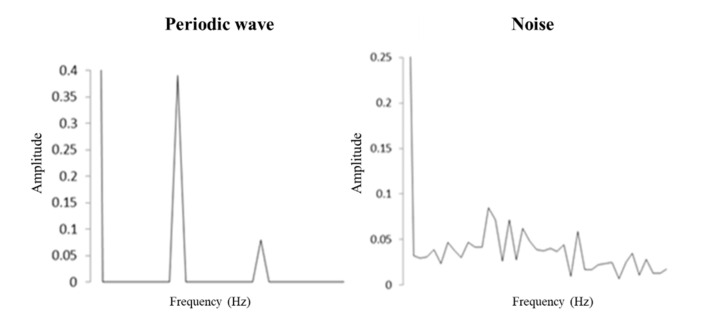
Visual illustration of Fast Fourier Transform analysis.

The FFT, which is a linear integral transformation approach, is used to process the segmented signals. The sum of sine functions is considered to measure the frequency of the continuous waves. Given the resource restrictions, the discretization of the results reduces the complexity of the operational analysis. The discretization process results in noise, which is shown as an irregular frequency, and periodic signals, which are shown as a regular frequency; Figure 5 is an illustrative example of this process.

To analyze the periodic signals, equipment availability is considered as the indicator. It indicates the operating time of the machines as a proportion of the total production time. This measure determines the efficiency of equipment usage and production operations as the key productivity parameters. Given the capital-intensive nature of the smart manufacturing systems, the return on investment is of paramount significance, which justifies the selection of the utilization rate as the performance indicator. Equipment availability, denoted by EA, is calculated using Equation (8). In this formulation, O represents the operating time of the actual equipment available and T is the total time of the equipment availability. This measure is used to compare the equipment availability found by the proposed monitoring approach and compare it with the actual value.

(8) $$EA=\frac{O}{T}$$

Once the different processing states are distinguished, they can be used for training the model. For this purpose, the HMM is applied to train the identified machining states and establish a model that aids the operator to quickly identify the current state of the production operations and take action if any anomalies are detected.

## 4. Experimental Results

**Figure 6:**
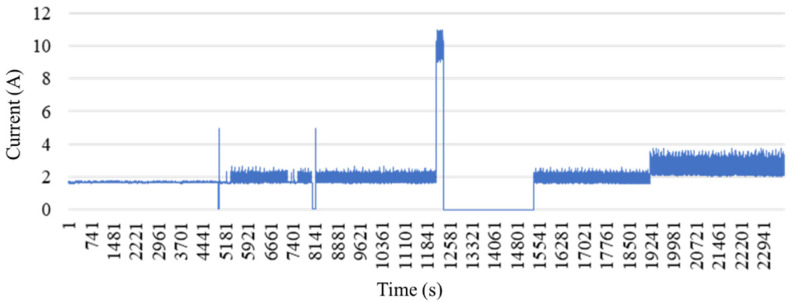
Experimental data.

This section evaluates the performance of the proposed smart monitoring framework that uses the output from non-invasive sensors connected to the machinery. The collected data is initially segmented and classified; the results are then used to identify the processing status, production performance, cycle time, and the availability of the equipment. Finally, the system establishes a trained classification to ensure the accuracy of the processing status and resolve the model. Real-world tests are used to evaluate the practicability of the developed framework; for this purpose, the following operational states are considered to determine the status of the equipment: standby, refueling, processing product A, breakdowns, stoppage, and processing product B. The experimental data obtained from the cutting machine is shown in Figure 6. Clamp-on current transformer is used to measure the current signal for monitor and record machine states. The source of the data is from the machine’s current signal. The proposed algorithm was implemented using the MATLAB.

**Figure 7:**
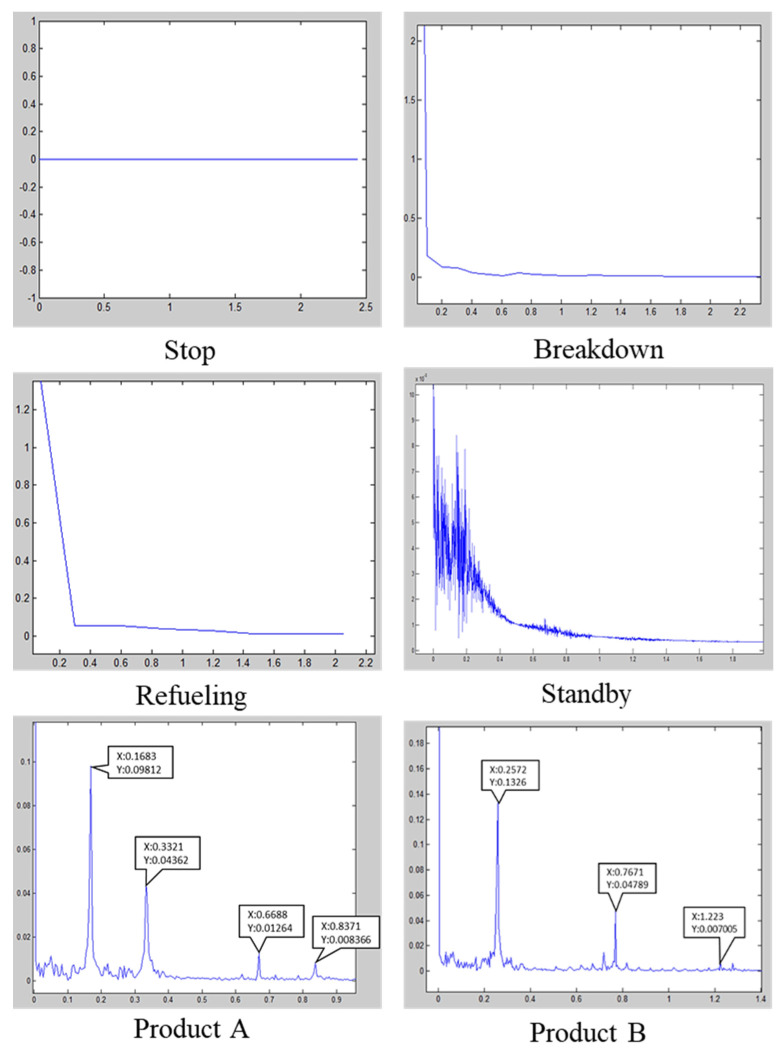
Fast Fourier Transform comparison in different states. (*x*-axes: Frequency(Hz); *y*-axes: Amplitude).

To begin with the experiments, *α* and *β* values are set to 6 and 15, respectively. Equations (6) and (7) are used to check whether the segments are processed; the segmentation continues until the required conditions are met. FFT determines the status considering the aforementioned conditions. Figure 7 presents the spectral characterization of each stage of the FFT. Since each product would have its different spectrum representing in amplitude and frequency. According to Figure 7, product A and product B have their spectra for identification. On this basis, the outcomes of the basic HHT segmentation are determined and presented in Table 1. The results show a significant difference in the standby and product A status when compared to the actual values. The reason for this deviation can be explained according to Figure 7, where the processing of a product can be confused by the standby status. In other words, the HHT segmentation method cannot effectively distinguish the non-processing status from the processing state due to the similarities in the respective signals. The error can be even more significant in more complex situations, resulting in a major operational deficiency.

The developed framework integrates the HMM with FFT to classify signals and construct a model for determining the machine status more efficiently and accurately. Signals from different processing states are used to verify the validity of the model. For this purpose, a total of twelve instances are considered to complete the experiments. The results are reported in Table 2 with the probability values representing the accuracy of the outcomes across the experiments.

The similarities in the test groups, which are highlighted in grey color, represent the accuracy of the outcome. The multi-method framework effectively determines the production status after training. However, the deviations observed in the signals associated with refueling and breakdowns show that more training data may be required for an accurate judgment of the system status. Equation (8) is used to determine the availability of the equipment as an OEE-based measure. Table 3 compares the actual and calculated values; it is shown that the results are very accurate with a negligible error of about 3.7%.

Next, the segmentation results are compared to that of the P-DTW method for calculating the processing cycle and the production quantity of products A and B. The results are summarized in Table 4. In this table, the processing time for products A and B was 5.8 and 4 s, respectively. P-DTW and processing cycle are considered to calculate the number of products. The number of product A items was 348, whereas that of product B was 222. For products A and B, the actual error rates are 0.87 and 1.77%, respectively, which are within an acceptable threshold. The analysis of the computational time and results confirm that the proposed method can provide the product line manager with timely and accurate feedback.

## 5. Concluding Remarks

The manufacturing sector has witnessed an exponential growth in the adoption of Industry 4.0 and its enabling technologies. PLCs have been widely implemented to collect the required operational data for real-time monitoring of the production processes. However, the environment, equipment type, cost-intensiveness, incompatibility with various machines and software limits the operational capabilities of PLCs. This study proposes a multi-method approach to contribute to the analysis of the data from noninvasive sensors for smart monitoring. Consisting of four major steps, noise elimination, signal segmentation, state classification, and manufacturing availability calculation, the proposed method enables intelligent decision-making in advanced manufacturing systems.

To demonstrate the applicability of the proposed approach, a real-world case with machine statuses like standby, refueling, failure, shutdown, and production is considered to conduct the numerical experiments. The objective was to determine the operating state and estimate the underlying performance, i.e., equipment availability and production quantities. The experimental results are supportive of the reliability of the proposed monitoring framework with less than 4 and 2 percentage of error for the determination of the availability and production states, respectively. In addition to reducing decision errors caused by missing or unreliable data, the computational efficiency of the proposed approach enables well-informed production planning and scheduling decisions based on real-time knowledge of the production performance. Overall, the proposed approach is viable for manufacturing-based operating systems if an appropriate training dataset is available.

The findings in this study can be used as a basis for the further development of intelligent decision-making in smart manufacturing systems. The following research directions are worthwhile to pursue for future studies. First, future research can extend our proposed approach to analyze the production yield rates through signals to identify the causes of failure and improve productivity. Second, the developed monitoring approach can be integrated into non-static decision aid frameworks, like dynamic capacity planning, to examine the impact of the parameter changes on the performance of the method. Finally, the Concept of Stratification and Incremental Enlargement can be deployed to help advance the fault-detection and alarming functions in smart monitoring.

## Figures and Tables

**Table 1:** Segmentation error obtained by Hilbert–Huang Transform (HHT) method.

Length Data	Standby	Refueling	Breakdown	Stop	Product A	Product B
HHT	4969	144	218	2999	10,905	4420
Actual	5538	149	219	2930	10,384	4435
Difference	569	5	1	69	521	15
Error (%)	10.3	3.4	0.5	2.4	5.0	0.3

**Table 2:** Processing status analysis of the multi-method framework.

Test	Status (%)						Result	Actual
	Standby	Refueling	Breakdown	Stop	Product A	Product B		
1	99	0	0	0	1	0	Standby	Refueling
2	99	0	0	0	1	0	Standby	Refueling
3	0	0	0	0	100	0	Product A	Refueling
4	0	0	0	0	100	0	Product A	Refueling
5	0	0	0	0	8	92	Product B	Breakdown
6	0	0	0	0	18	82	Product B	Breakdown
7	0	0	0	100	0	0	Stop	Stop
8	0	0	0	100	0	0	Stop	Stop
9	0	0	0	0	80	20	Product A	Product A
10	0	0	0	0	78	22	Product A	Product A
11	0	0	0	0	11	89	Product B	Product B
12	0	0	0	0	16	84	Product B	Product B

**Table 3:** Performance analysis of the system.

As-Is Availability (%)	Calculated Availability (%)	Error (%)
71.5	74.2	3.77

**Table 4:** Analysis of the computational time and results.

Product	Computational Time (s)	Quantity		Error	
		Actual	Calculated	Absolute	Rate (%)
A	5.8	345	348	3	0.87
B	4	226	222	4	1.77
